# To glide or to swim? A reinvestigation of the enigmatic *Wapitisaurus problematicus* (Reptilia) from the Early Triassic of British Columbia, Canada

**DOI:** 10.1098/rsos.231171

**Published:** 2023-11-15

**Authors:** Dylan Bastiaans, Valentin Buffa, Torsten M. Scheyer

**Affiliations:** ^1^ Palaeontological Institute and Museum, University of Zurich, Zurich 8006, Switzerland; ^2^ Evolutionary Studies Institute, University of the Witwatersrand, Private Bag 3, Johannesburg, Gauteng WITS 2050, South Africa; ^3^ Muséum National d'Histoire Naturelle, Centre de Recherche en Paléontologie – Paris, UMR 7207 CNRS-MNHN-SU, CP38, 8 Rue Buffon, Paris, Île-de-France 75005, France

**Keywords:** Early Triassic, Wapiti Lake, Spathian, Thalattosauria, Weigeltisauridae, *Wapitisaurus*

## Abstract

*Wapitisaurus problematicus* was initially described as a member of the Weigeltisauridae, a clade of Late Permian gliding reptiles from Eurasia and Madagascar. However, the poor preservation of the holotype and only known specimen, from the lower Sulphur Mountain Formation at Ganoid Ridge (British Columbia, Canada), raised doubts about this assignment. Here, we redescribe *W. problematicus* and reassess its systematic position among diapsid reptiles. Comparison with all known weigeltisaurids, as well as contemporaneous reptiles from the Sulphur Mountain Formation, indicates that the taxon instead represents a thalattosauroid thalattosauriform, with noted similarities to *Thalattosaurus* and *Paralonectes*. This reidentification restricts weigeltisaurids to the Late Permian, with no occurrence in North America. *Wapitisaurus problematicus* potentially represents one of the oldest thalattosauriforms and increases our understanding of their diversity and disparity during the late Early and Middle Triassic. The close morphological similarities with later (thalattosauroid) thalattosauriforms and their high abundance in (shallow) marine settings may indicate an earlier invasion of this realm than previously assumed. This parallels observations in early ichthyopterygians with widespread opportunistic trophic niche diversification occurring relatively rapidly after the end-Permian mass extinction event.

## 1. Introduction

The enigmatic reptile *Wapitisaurus problematicus* [1] presumably from the Early Triassic of Ganoid Ridge, British Columbia, Canada was described as a putative large-sized member of the Weigeltisauridae, a group otherwise composed only of small late Permian gliding reptiles [1]. However, the large size and poor preservation of the holotype and only known specimen of *W. problematicus* (TMP 86.153.14) has led authors to exclude this taxon from considerations on weigeltisaurid comparative anatomy and evolution [2–4], with some going even as far as to suggest that this taxon is not a weigeltisaurid [5].

The initial description of *W. problematicus* as a potential member of Weigeltisauridae, previously absent from the North American fossil record, was based on comparison with limited material of the—then only known—weigeltisaurid genus *Coelurosauravus* from western Europe and Madagascar [1]. Similarities of the triangular skull shape with a large orbit, and a purportedly completely preserved postorbital region with ‘incomplete lower temporal arcade, a jugal with reduced postorbital process, and a squamosal crest ornamented with tooth-like projections' (see [1, p. 951]) were noted as shared features between the new taxon and *Coelurosauravus*. Based on the larger size of TMP 86.153.14, that study did not consider *W. problematicus* necessarily as a glider, contrary to all other weigeltisaurids, without further speculating about its palaeoecology and lifestyle. In addition, differences with *Coelurosauravus* in tooth shape and mode of tooth implantation observed in the dentary bone were noted as the basis for erecting a new species and genus of Weigeltisauridae. Similarities with the (sub)thecodont tooth implementation found in thalattosaurs and some ichthyosaurs were also pointed out, but aspects of the purported general skull shape, especially of the postorbital region and temporal fenestra shape, which were considered to be complete and *in situ*, were used to argue against closer relationships with both marine reptile groups [1].

However, much of the cranial anatomy of weigeltisaurids has since been revised in light of new fossil discoveries [2,6–8], and re-description of historical specimens [3,4,9–11]. These new data offer a more detailed reconstruction of the weigeltisaurid skull that is markedly different than what was believed at the time of the description of *W. problematicus* [12,13], most notably in the possession of a single, very large temporal fenestra framed ventrally by a fully closed infratemporal bar [3,4,9,10].

It is also noteworthy that in 1988, most of the thalattosaur material from Ganoid Ridge was not yet recovered and would be formally described only five years later [14], leading to the erection of three new thalattosaur species, *Agkistrognathus campbelli*, *Paralonectes merriami* and *Thalattosaurus borealis*. These three species are all still considered to be valid species of the Thalattosauroidea within Thalattosauria [15]. A later revision of the North American *Thalattosaurus* and *Nectosaurus* material from the Late Triassic Hosselkus Limestone of California [16] also did not include a discussion of *W. problematicus* as there was no reason to doubt its affinities at the time given the then presumed understanding of the weigeltisaurid cranium.

In this context, we re-analyse TMP 86.153.14, the holotype and only known specimen of *W. problematicus*, in light of novel data that have recently become available on both, Weigeltisauridae and Thalattosauria. We especially highlight the comparison with the holotype material of the thalattosauroid *P. merriami*. We argue that the overall shape of the skull of *W. problematicus* is not as easily reconstructed as previously indicated as most bones of the lower temporal arcade, posterior skull table and occipital region are missing. Given this lack of purportedly shared features with *Coelurosauravus*, the general anatomy of *W. problematicus* is more consistent with that of thalattosauroid thalattosaurs instead.

## 2. Material and methods

### 2.1. Institutional abbreviations

**MBR**: Museum für Naturkunde Berlin, Invalidenstrasse 43, 10115 Berlin, Germany;

**MNHN**: Muséum National d'Histoire Naturelle, 57 Rue Cuvier, 75005 Paris, France;

**PIMUZ**: Paleontological Institute and Museum, University of Zurich, Karl-Schmid-Strasse 4, 8001 Zurich, Switzerland;

**PIN**: Paleontological Institute of the Russian Academy of Sciences, Ulitsa Profsoyuznaya 123 Moscow, Russia;

**(R)TMP**: (Royal) Tyrrell Museum of Paleontology, PO Box 7500, Drumheller, Alberta, Canada T0J 0Y0;

**SMNK**: Staatliches Museum für Naturkunde Karlsruhe, Erbprinzenstrasse 13, 76133 Karlsruhe, Germany;

**SSWG** : Sektion Geologie, Ernst-Moritz-Arndt Universität, Domstrasse 11, 17489 Greifswald, Germany;

**UCMP**: University of California Museum of Paleontology, Berkely, California 94720-4780, USA.

### 2.2. Materials

**Figure 1.**
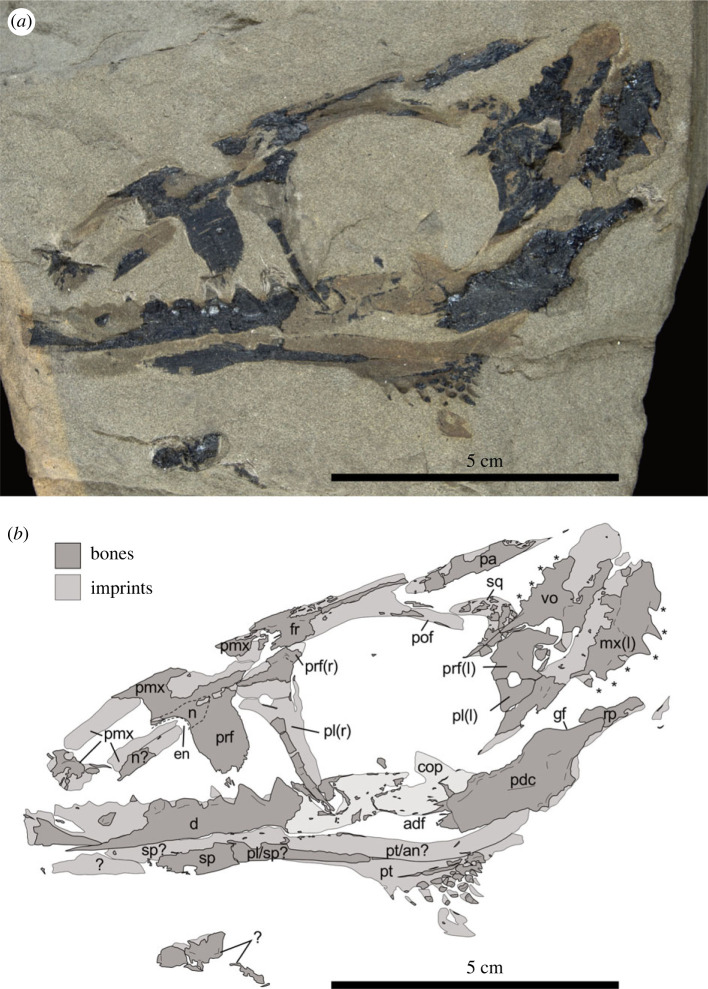
Photograph (*a*) and interpretative sketch (*b*) of the holotype TMP 86.153.14 of *Wapitisaurus problematicus* [1]. The fossil consisting of bone fragments and bone imprints shows mostly the right side of the skull and mandible in internal view if not indicated otherwise (l = left side; r = right side). **adf**, adductor fossa; **an**, angular; **cop**, coronoid process; **d**, dentary; **en**, external naris; **fr**, frontal; **gf**, glenoid fossa; **mx**, maxilla; **n**, nasal; **pa**, parietal; **pdc**, postdentary complex (incl. articular, surangular and potentially aspects of prearticular and angular); **pl**, palatine; **pmx**, premaxilla; **prf**, prefrontal; **pof**, postorbitofrontal; **pt**, pterygoid; **rp**, retroarticular process; **sp**, splenial; **sq**, squamosal; **vo**, vomer; *, marginal or palatal dentition.

The holotype specimen TMP 86.153.14 (figure 1*a,b*) consists of a very fragmentarily preserved skull and mandible, found in scree material presumably from siltstone layers of the Vega-Phroso Member of the Sulphur Mountain Formation at Ganoid Ridge, Wapiti Lake area, British Columbia, Canada [1]. The age of the specimen was at the time of its first description considered to be Smithian (lower Olenekian, Early Triassic), which would make it one of the oldest reptiles recovered from the Wapiti Lake area. Fortunately, the exact coordinates of the specimen were given as ‘UTM 647 000 E., 6 045 000 N., zone 10, map 93 I/10’ (see [1, p. 952]), which, converted, correspond to a latitude of 54.531201° N (=54°31′52.3″ N) and a longitude of 120.728209° W (=120°43′41.6″ W), about 5 km south-southeast of Wapiti Lake. The location could thus be identified as lying at cirque B, close to the highly fossiliferous cirque C that yielded a rich fish fauna from Ganoid Ridge [14,17].

The fish fossils recovered from cirque C have been dated to the early Smithian based on conodont biostratigraphic zones with the presence of *Conservatella conservativa* and *Neospathodus cristagalli* [17,18]. Owing to the complex geology including the progression of a major anticline in the area ([19]; see also [20]) that probably affects the exposure of sediment layers in cirques A, B and C, and the absence of conodonts associated with TMP 86.153.14, a younger, Spathian age cannot be ruled out. The lithology of TMP 86.153.14 is inconsistent with that of the overlying Whistler Member, however, an assignment to the Middle Triassic Llama Member, as suggested by Neuman [21], cannot be fully excluded. Nonetheless, the lack of associated invertebrate material such as high concentrations of fragmented shells and the absence of bioturbation, both ubiquitous in the Llama Member [21], may provide additional support for an assignment to the Vega-Phroso Siltstone Member. However, further geochemical, sedimentological and microfossil analyses are required to unequivocally determine the source member of *W. problematicus*.

Other presumed Lower Triassic thalattosaur material has been recovered from ‘cirque N’ (e.g. TMP 1983.218.7, TMP 1991.117.8) at Wapiti Lake and from the Smithian (?) of Meosin Mountain (e.g. TMP 1996.72.20, TMP 1996.72.31, TMP 1996.72.1, TMP 1996,72.28, TMP 1996.72.12, TMP 1996.72.21, TMP 1996.72.2), some of which may represent the genera *Paralonectes* and/or *Agkistrognathus*.

The thalattosaur material recovered later from Ganoid Ridge from cirque T (including the holotype of *T. borealis*, TMP 89.126.1) was indicated to be mid-Triassic in age, and that of cirque D (including the holotype TMP 89.127.1 of *P. merriami*, and of holotype TMP 89.127.6 of *A. campbelli*), either Early or Middle Triassic [14]. It is noteworthy that one very incomplete specimen of *P. merriami* (TMP 91.120.21) was also recovered from ‘cirque B’ in 1991, although the provided coordinates for the material (54°31'10″ N and 120°43'19″ W; [14] indicate only the ‘cirque D’ (see [18]: figure 1).

## 3. Results

### 3.1. Systematic palaeontology

Thalattosauria Merriam, 1904 [22]

Thalatosauroidea Nopcsa, 1928 [23]

*Wapitisaurus problematicus* Brinkman, 1988 [1]

Horizon and locality: siltstone layers of the Vega-Phroso Member of the Sulphur Mountain Formation in Cirque B at Ganoid Ridge, Wapiti Lake area, British Columbia, Canada (54.531201° N; 120.728209° W).

Revised diagnosis: *W. problematicus* can be distinguished from other thalattosauroids on the basis of a unique combination of derived characters, including: its size, representing a 1- to 2 m long thalattosaur; premaxillae with very long supranarial processes that contact the frontal, excluding the nasals from the midline along the skull roof and precluding internasal contact; short premaxillae with modest ventral deflection, perhaps not extending below the marginal tooth row; robust vomer with short posterior process and at least four teeth; maxillae with long posterior (jugal) process and short dentigerous margin, terminating well anterior to or at the orbital rim, each bearing six sharp teeth with slightly recurved apices and anteriorly upturned tooth row; external naris posteriorly located, shortly in front of orbit; nasal is complex, probably L-shaped and anteriorly extensive; relatively small prefrontal and squamosal; an unusually large orbit, perhaps up to one-third of anteroposterior cranial length; a restricted posterior (postorbital) cranial length; posterior mandibular dentition shows a larger occupation along the medial dental shelf; a prominent coronoid process, with a long anterior and shorter posterior process, projecting dorsally or slightly posterodorsally; deep glenoid fossa with thickened margins; a robust, well-developed and anteroposteriorly extensive pterygoid with numerous large teeth on transverse process, comprising roughly two-thirds of the anteroposterior orbital length; marginal and mandibular dentition set in prominent sockets, implantation is (ankylosed) thecodont; as well as autapomorphic traits including a (presumed) very short postorbital region; a relatively straight mandibular shelf that terminates in a short horizontally oriented retroarticular process; mandibular dentition consisting of at least 11 to 12 sharp conical teeth on each side and with only a minor degree of heterodonty with respect to size and shape.

### 3.2. Reinterpretation of skull and mandible osteology

**Figure 2.**
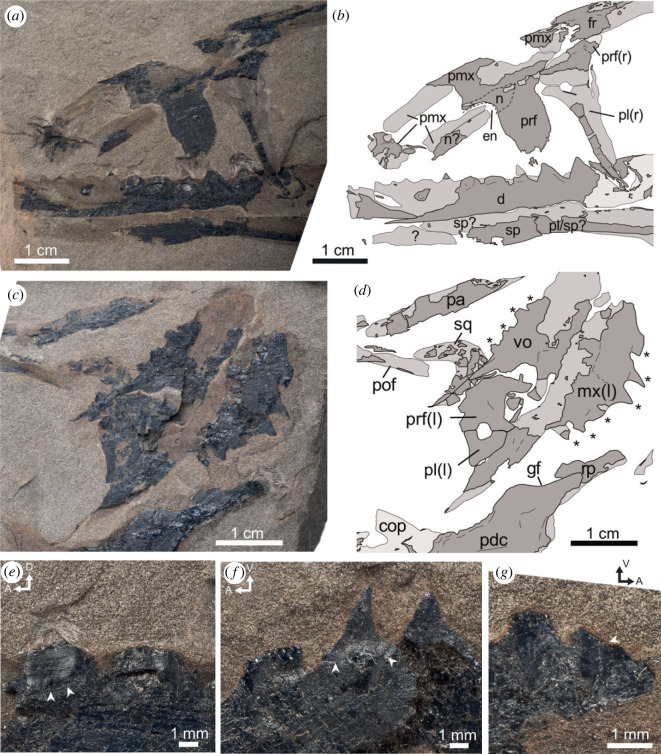
Close up photographs and interpretative sketch of rostral (*a,b*) and postorbital (*c,d*) regions, and dentary (*e*), maxillary (*f*), and vomerine (*g*) teeth of the holotype TMP 86.153.14 of *Wapitisaurus problematicus* [1]. The fossil consisting of bone fragments and bone imprints shows mostly the right side of the skull and mandible in internal view if not indicated otherwise (l = left side; r = right side). Arrowheads indicate the separation between the tooth and bone of attachment. Arrows indicate anterior (A), dorsal (D), and ventral (V) direction. **cop**, coronoid process; **d**, dentary; **en**, external naris; **fr**, frontal; **gf**, glenoid fossa; **mx**, maxilla; **n**, nasal; **pa**, parietal; **pdc**, postdentary complex; **pl**, palatine; **pmx**, premaxilla; **pof**, postorbitofrontal; **prf**, prefrontal; **pt**, pterygoid; **rp**, retroarticular process; **sp**, splenial; **sq**, squamosal; **vo**, vomer; *, marginal or palatal dentition.

The holotype of *W. problematicus* TMP 86.153.14 only comprises a partial skull and right lower jaw (figure 1). Parts of the right side of the rostrum and skull roof are preserved in medial view while the left side is mostly missing. By contrast, [1] interpreted the available material as part of the left side of the skull. Elements from the palate overlie the right half of the skull, some of which have been subject to postmortem rotation and displacement (figure 2). Lastly, the left maxilla has been strongly displaced and rotated so that it is visible in medial view, overlying other preorbital skull bones. The available bones are themselves rather poorly preserved and are represented by bone fragments and imprints in the matrix. As follows, the re-examination of the cranial remains of *W. problematicus* (figure 1*a,b*) has resulted in a slightly divergent interpretation of the elements represented in TMP 86.153.14 compared to the original description [1].

The premaxilla of *Wapitisaurus* is largely missing and predominantly preserved as an impression in right medial view (figure 2*a,b*). The alveolar margin of the premaxilla is not preserved so that no tooth socket or teeth can be seen. The premaxilla is elongated with a slight ventral curvature anteriorly. The bone has a very long supranarial process that reached the frontal posteriorly, the latter of which forms a concavity for accepting the former (figure 2*a,b*). As the anterior portion of the premaxilla is badly broken, it is unclear whether it participated in the dorsal margin of the external naris. A slender, elongate fragment lying slightly more ventrally may represent a fragment of the subnarial process or anterior process of the nasal, although this cannot be ascertained.

The premaxilla overlies a wide plate-like bone structure forming part of the right lateral surface of the cranium anterior to the orbit. The anterior and posterior margins of this structure are subvertical. However, the anterodorsal margin of the bone is markedly concave. Given that this margin is smooth and shows no trace of breakage, we identify it as the posterodorsal margin of the external naris. Under this interpretation, this plate-like structure is probably formed by the nasal, bearing the preserved portion of the external naris, the prefrontal and possibly some portion of the frontal posterodorsally. Although the suture between these bones is hard to distinguish, the nasal appears to constitute only a small, crescentic portion of this structure around the external naris while the massive prefrontal forms a wide, plate-like contribution to the antorbital rostrum. In addition, a slender, elongate fragment associated with the putative subnarial process of the premaxilla may represent an anterior process of the nasal that was broken off and slightly displaced. Probably an elongated anterior process of the nasal precluded the supranarial premaxillary process from accessing the external naris, as observed in other North American thalattosaurs (e.g. *Thalattosaurus*, *Nectosaurus*) [16,24]. Thus, the nasal may have been elongate, and formed the entire posterior and dorsal margins of the external naris. Lastly, the external naris is located rather close to the orbit, separated from the anterior margin of the skull by a long premaxillary rostrum. On the dorsal skull roof, behind the premaxilla, there is an elongate element with strongly concave anterior and posterior margins that bear median and lateral prong-like processes (figures 1 and 2). This element represents the frontal bone. Anteriorly, the frontal would have accommodated the posterodorsal process of the maxilla laterally, with the anteromedial process of the frontal presumably separating the premaxilla and the nasal. The anterolateral process of the frontal abuts the prefrontal laterally, and presumably the nasal with its anteromedial portion. It would have probably been precluded from contacting the external naris by the contact between the nasal and premaxilla for its medial process, and the nasal and posterodorsal process of the maxilla for its lateral process. Based on what is preserved of the prefrontal and postorbitofrontal, the frontal probably moderately participates in the dorsal margin of the orbit. More posteriorly, the frontal articulated with the parietal, the latter of which lies posteriorly and slightly ventral to the caudal margin of the frontal.

Based on the imprint of the frontal, its posterior margin appears strongly concave between its posteromedian and as preserved, its longer posterolateral processes, which suggests that the frontoparietal suture was crescent-shaped.

The parietal is very incomplete, precluding a detailed anatomical description (figure 2*c,d*). Based on its preserved portion, and on the general proportion of the skull and mandible, it was probably slightly anteroposteriorly shorter than the frontal.

The postorbitofrontal is only represented by an impression projecting along the posterodorsal border of the orbit, adjoined to the posterolateral process of the frontal (figures 1 and 2). It appears slightly displaced posteroventrally, creating the impression of a more anteroposteriorly extensive orbit.

Posterior to the postorbital, there is a short, anteriorly concave, hook-like imprint that appears to link several small bone fragments. Given the shape of this structure, we interpret it as the posterior part of the right squamosal visible in medial view. The bone appears to comprise slender anterior and ventral processes, both of which are missing their distal extremities, set at an acute angle to each other. The posterodorsal border of this concavity shows some scalloping, and a thickening of the element, probably accommodating the quadratic condyle. Dorsally, the element is relatively straight and may have been more extensive anteriorly. Distally the element terminates in a somewhat sharp tip that is overlain by a large element.

As discussed above, the left maxilla and some elements of the anterior palate have undergone strong postmortem rotation and displacement and now overly the right side of the skull, particularly its postorbital region (figures 1 and 2). Indeed, the ‘tooth-like projections' (see [1, p. 953]) on the large element that [1] tentatively identified as an ornamented squamosal are actually very similar in shape and texture to the unambiguous marginal teeth of the right lower jaw, albeit slightly smaller and slightly posterodorsally recurved instead of (antero)dorsally as in the mandible (figure 2). Here, we interpret these projections as maxillary teeth, indicating that the left maxilla lies in medial view, positioned just above the posterior end of the lower jaw. Although the outline of the bone is hard to follow, the ventral margin of the bone is mostly preserved, missing only its anteriormost part. By contrast, the bone is mostly incomplete dorsally, although we suggest that part of the dorsal process of the maxilla is preserved, forming a jumbled and poorly preserved bony mass overlying the left prefrontal and palatine (figure 2*d*; see below). Six sharp teeth can be identified, indicating a closely spaced dentition. The anterior teeth are large, sharp and conical. They slightly increase in size up to the third tooth position, after which tooth size progressively decreases. The marginal dentition terminates well before the posterior end of the jugal process of the maxilla. Approximately half of the preserved maxillary length is edentulous. The crowns seem to be upturned along the anterior margin of the maxilla and slightly dorsally displaced posteriorly, giving the dentition an overall wave-like apical margin.

Similar to the left maxilla of [1], we also interpret the ‘projections’ on the bone previously identified as the supratemporal, as teeth. However, despite incomplete preservation they differ from the upper and lower marginal dentitions in their smaller size, more posteriorly placement and presumed blunt shape (although notably incomplete). Thus, we interpret them as palatal teeth, and the corresponding bone as an unpaired, massive vomer that underwent strong displacement and rotation so that it lies just dorsal to the left maxilla, partially overlying the right squamosal with its short posterior process (figure 2*c,d*). Incidentally, similarly severely displaced massive vomers can also be seen in some thalattosaurs from the Wapiti Lake region (e.g. figs 2 and 9 in [14] and TMP 96.72.1, TMP 1991.122.10). The vomer is a robust element, being only slightly shorter than the preserved portion of the left maxilla. The posterior portion of the bone appears mostly preserved while its anterior portion is visible only as an imprint. The anterior margin of the vomer appears relatively round or blunt. Posteriorly, the bone bears a thin long process that probably formed a concave, V-shaped posterior margin. The vomer bears four partial teeth, with perhaps an impression of another two alveoli anteriorly. The dorsal margin appears relatively straight for most of its length, only slightly rounding off anteriorly and sharply declining in height posteriorly.

Anterior to the orbit, there is an L-shaped element that extends ventrally from the anterior region of the frontal to the level of posterior end of the dentary tooth row, which it partially overlays. Given this location, we suggest that this element may represent the right palatine that underwent postmortem rotation (figure 2*a,b*). The palatine tapers to a point posteriorly and has relatively straight anterior and ventral margins. Anteriorly the dorsoventrally extensive margin would have contacted the prefrontal along the ventromedial margin of the latter. Although the thickness of the bone is hard to assess given its preservation, the marked distinction between its slender medial margin, preserved as bone, and its wider lateral margin, preserved as an imprint, may suggest that the palatine is narrower medially than laterally. This suggests that the palatine is slightly transversally concave, with a raised lateral margin bracing against the medial surface of the maxilla and a robust but less dorsoventrally extensive medial margin meeting the other palatine. Lastly, a shallow break in the anteriormost part of the bone may represent a fragment of the prefrontal which remains in articulation, although this is difficult to ascertain.

Along the posterior orbital margin and anterior to the preserved maxilla and vomer lies a broken tear-drop-shaped jumble of bones. As mentioned above, this structure probably comprises part of the dorsal process of the left maxilla, as well as the left prefrontal and palatine (figure 2*c,d*). The latter two bones would form the anteriorly concave margin seen between the posterior processes of the maxilla and that of the vomer. However, the sutures between these bone fragments have been obliterated by postmortem compression. Based on the close association between the purported palatine portion of this structure and the left maxilla, it is likely that the palatine had an extensive contact with the ventromedial maxilla, perhaps along a medial shelf, the border of which may be faintly illustrated by a thickening along the dorsal margin of the posterior maxillary process.

The left pterygoid is largely preserved as an impression ventral to the mandible. More than 18 teeth can be observed, most of which are tooth bases with broken-off crowns. However, several retain their sharp recurved conical apexes. The teeth situated in the central region of the pterygoid appear to be the largest, as evidenced by the dimensions of their tooth bases. The teeth on the pterygoid appear to be arranged in several (≥5) roughly posteriormedially oriented rows and are restricted to the region of the pterygoid shelf. The dorsal surface of the shelf seems to have been concave, with a thickened lateral margin and a slightly taller medial margin. Posterior to the transverse process of the pterygoid, the base of the quadrate process is partially preserved as a dorsoventrally tall bar-like impression.

There are additional fragments and impressions of bones seen ventral to the pterygoid and mandible; however, these are too poorly preserved to be identified with certainty.

The right mandible of *Wapitisaurus* is largely preserved, although some anterior and posteroventral sections may be missing. Ten to twelve sharp and laterally compressed, conical teeth with wide bases and slightly anteriorly curving apexes, are preserved along the straight dorsal margin of the dentary. These teeth are closely spaced, are roughly equally wide as tall and seem to touch at their bases. The mode of tooth implantation appears to be (sub)thecodont (*sensu* [25]), as indicated by the tall expansion of the labial wall over the tooth root (figure 2*a,b*). The teeth show a strong attachment to the jawbone, suggesting an ankylosis type of tooth attachment. The anterior dentition is slightly shorter, smaller and more conical in appearance compared to the rest of the teeth, based on impressions. The dentition extends posteriorly until the anterior border or within the anterior quarter of the orbit, with the posterior half of the mandible being edentulous. In its posterior quarter of the mandible, there may be a slight degree of dorsal flexure of the dorsal mandibular margin. However, it is impossible to rule out postmortem deformation creating this flexure, given the displaced and rotated elements in the posterior skull region, such as the parietal, squamosal, left maxilla and antorbital elements.

A faint impression of a well-developed coronoid process can be observed in the posterior half of the orbit. The outline of the coronoid bone can be mostly followed as an imprint. The coronoid has a wave-like outline, with a depressed anteroventral, and raised posterodorsal and dorsal extent. The anteroventral process is the most extensive of the three, projecting as far anteriorly as the coronoid process is tall and terminating in a blunt or slightly rounded anterior margin. The dorsal margin of the coronoid is concave posteriorly, whereas the dorsal process extends relatively straight towards the orbit. The ventral margin of the coronoid is difficult to discern but probably is sinusoidal, being slightly convex up until the level of the dorsal process, after which it becomes concave and posterodorsally directed, terminating in a slightly rounded or tapered end. The posterior margin of the coronoid process is difficult to assess based on the preserved impressions but may have been concave and straightening out anterodorsally. The coronoid process, as preserved in medial view, is approximately a quarter longer anteroposteriorly than it is tall, with only about a third exposed above the dorsal margin of the mandible. The portion that extends above the mandibular shelf in life as seen from lateral view would have been roughly twice as anteroposteriorly long as dorsoventrally tall. The posterodorsally pointed apex of the coronoid matches the wave-like shape of the ventral margin. The anterior process no longer touches the dorsal margin of the mandible. This displacement is probably postmortem and seems related to fracturing observed in that portion of the mandible. This displacement would also explain the posterodorsal angle of the posterior mandible, including the articular and retroarticular process. An impression of the splenial may be preserved ventral to the anterior dentary, overlapping with the pterygoid and perhaps a palatine portion. This elongated and anteriorly pointed element probably would have overlapped the angular posteriorly, although clear sutural contacts cannot be distinguished.

The posterior mandible is too poorly preserved to identify sutural contacts. A glenoid fossa of the articular can be identified, terminating at the level of the posterior-most left maxillary tooth. This fairly restricted depression is surrounded by thickened bone. A well-developed mandibular condyle of the quadrate would have been partially sunk beneath the dorsal mandibular margin in articulation. Anterior to the glenoid fossa, the mandible seems to display a slightly raised dorsal margin. At the posterior end, a short retroarticular process can be identified, which is equal in length to the anteroposterior extent of the glenoid fossa.

## 4. Discussion

### 4.1. Taxonomic identification of *Wapitisaurus problematicus*

In his initial classification of *W. problematicus* as a weigeltisaurid, [1, p. 953] cited the following shared characteristics: ‘ornamented squamosal, incomplete lower temporal arcade, and jugal with reduced posterior process'. However, our detailed re-examination of TMP 86.153.14 demonstrated that the jugal is not preserved in this specimen, and that the ‘tooth-like ornamentations’ identified by [1] actually represent palatal and marginal teeth, allowing for the reidentification of the purported ‘squamosal’ and ‘supratemporal’ as the left maxilla and the vomer, respectively. Moreover, new fossil discoveries [2,6–8] and revisions of historical material [3,4,9–11] have since demonstrated that all weigeltisaurids actually have a closed infratemporal bar formed in most part by a long posterior process of the jugal. Consequently, the shared characters between *W. problematicus* and weigeltisaurids considered by [1] are no longer valid. Furthermore, as also noted by [1], the marginal dentition of TMP 86.153.14 consists of few (sub)thecodont conical teeth that are as tall as their base is wide. By contrast, all weigeltisaurids exhibit numerous, slender pleurodont marginal teeth [2–4,8]. In addition, the pterygoid teeth of weigeltisaurids are extremely small compared to that of TMP 86.153.14 [3]. Lastly, the cranial length of *Wapitisaurus* is probably over 100 mm, which is remarkably large compared to the smaller sizes of weigeltisaurids (i.e. *Coelurosauravus*: 36 mm, *Weigeltisaurus*: 51–57 mm, and *Glaurung*: 60 mm; tables 1 and 2; see [1,3,4,9]). In the light of these differences, we find little to no support for a weigeltisaurid identification of *W. problematicus*. Further comparison between weigeltisaurids and *Wapitisaurus* is listed in tables 1 and 2.

**Table 1.** A comparison of the cranial and mandible morphology of *Wapitisaurus problematicus* (TMP 86.153.14) to coeval thalattosauroids. (Note the similarities in cranial and dental features.)

species	*Thalattosaurus borealis*	*Paralonectes merriami*	*Agkistrognathus campbelli*	*Wapitisaurus problematicus*
specimens studied	TMP 89.126.1	TMP 89.127.1	TMP 89.127.6	TMP 86.153.14
		TMP 89.127.2		
skull size	>100 mm	>100 mm	>100 mm	>100 mm
pterygoid shape	massive	massive, slightly curved	unknown	massive, slightly curved
pterygoid teeth	multiple tooth rows, all broken off	multiple tooth rows, all broken off	unknown	multiple tooth rows, conical teeth
squamosal shape	crescent-shaped		unknown	unknown/crescent shaped
squamosal horn-like bony projections	absent	absent	unknown	unknown
dentary shape/depth	massive		massive, deep	massive, deep
dentary teeth count	>7 according to Nicholls and Brinkman, 1993 its 15–16	13–15	15	11–12 estimated as preserved
dentary teeth shape	P to A: bulbous/globular to more conical; rounded crowns	P to A: 5 to 7 teeth triangular, followed by 4–5 smaller teeth; at least 3 anterior-most teeth conical and enlarged again	P to A: posterior 6 teeth triangular and larger, followed by 5–6 smaller conical teeth, anterior 4 teeth conical and enlarged again	posterior 6 teeth triangular and pointed, followed by at least 5 anterior tooth impressions of unknown height
maxillary teeth count	unknown	6–7	7	unknown/ >5–6
data	this paper; [14]	this paper; [14]	this paper; [14]	this paper; [1]

**Table 2.** A comparison of the cranial and mandible morphology of *Wapitisaurus problematicus* (TMP 86.153.14) with coeval weigeltisaurids. (Note the clear differences in cranial length, tooth count and crown morphology.)

species	*Coelurosauravus elivensis*	*Weigeltisaurus jaekeli*	*Glaurung schneideri*	*Rautiania* spp.	*Wapitisaurus problematicus*
specimens studied	MNHN.F.MAP317a, b	SSWG 113/7	MBR, no. 3610 (cast)	PIN No. 5130/4	TMP 86.153.14
	MNHN.F.MAP325a	SMNK-PAL 2882	PIN No. 5392/1 (cast)	PIN No. 5130/62	
skull size	ca. 36 mm	ca. 51 / 57 mm	ca. 60 mm	unknown	>100 mm
pterygoid shape	slender, strongly curved	unknown	unknown	unknown	massive, slightly curved
pterygoid teeth	rows of tiny denticles	unknown	unknown	unknown	multiple tooth rows, conical teeth
squamosal shape	crescent-shaped	crescent-shaped	crescent-shaped	crescent-shaped	unknown/crescent shaped
squamosal horn-like bony projections	present		present	present	unknown
dentary shape/depth	slender, elongated	slender, elongated	slender, elongated	unknown	massive, deep
dentary teeth count	unknown	32	<25 estimated	unknown	11–12 estimated as preserved
dentary teeth shape	unknown	small, peg like with crowns slightly recurved	small, peg like with asymmetrical and slightly expanded crowns	unknown	posterior 6 teeth triangular and pointed, followed by at least 5 anterior tooth impressions of unknown height
maxillary teeth count	>18	21–23	unknown	23 / 30	unknown/ >5–6
data	this paper; [3]	[2–4,8–11]	[8–11]	[2]	this paper; [1]

Overall, our interpretation of the antorbital morphology of TMP 86.153.14 differs markedly from that of most early reptiles in the presence of a very long supranarial process of the premaxilla reaching the frontal, a posteriorly placed external naris framed by a slender, crescentic nasal. Indeed, in most non-saurian diapsids (e.g. *Claudiosaurus*, *Youngina*; [26,27]), and early saurians (*Clevosaurus*, *Protorosaurus*, *Prolacerta*; [28–30]), the premaxilla is restricted to the anterior extremity of the rostrum, with the external naris located very anteriorly on the skull, and the nasal is platelike, forming a significant portion of the lateral surface of the rostrum. Weigeltisaurids also share this general morphology, although they show a short premaxillary rostrum [4]. However, the morphology exhibited by TMP 86.153.14 would conform well to the rostral morphology of thalattosaurs, as best seen in *Thalattosaurus alexandrae* (fig. 1 in [16]), which all share a long premaxillary rostrum, slender nasal and posteriorly located external naris.

Conversely, [1] also noted some similarities between *W. problematicus* and thalattosaurs and ichthyosaurs, the two reptile groups that are most represented in the Early-Middle Triassic deposits at Wapiti Lake. In *W. problematicus*, the very long supranarial process of the premaxilla reaches the frontal, excluding the nasal from the skull roof midline and precludes contact between the nasals. By contrast, the nasal separates the premaxilla from the frontal in early ichthyopterygians, including those found in the Wapiti Lake region (*Gulosaurus, Utatsusaurus*; [31,32]). Furthermore, *W. problematicus* possesses numerous large teeth on the transverse process of the pterygoid. By contrast, most ichthyosaurs lack pterygoid dentition [33], with potentially only ‘vestigial’ pterygoid teeth being present in stem ichthyosauriform *Utatsusaurus* [34]. However, to ascertain the presence of denticles on the pterygoid in that taxon more evidence is required. Based on these two characters, we thus find it unlikely that *W. problematicus* is an ichthyopterygian.

**Figure 3.**
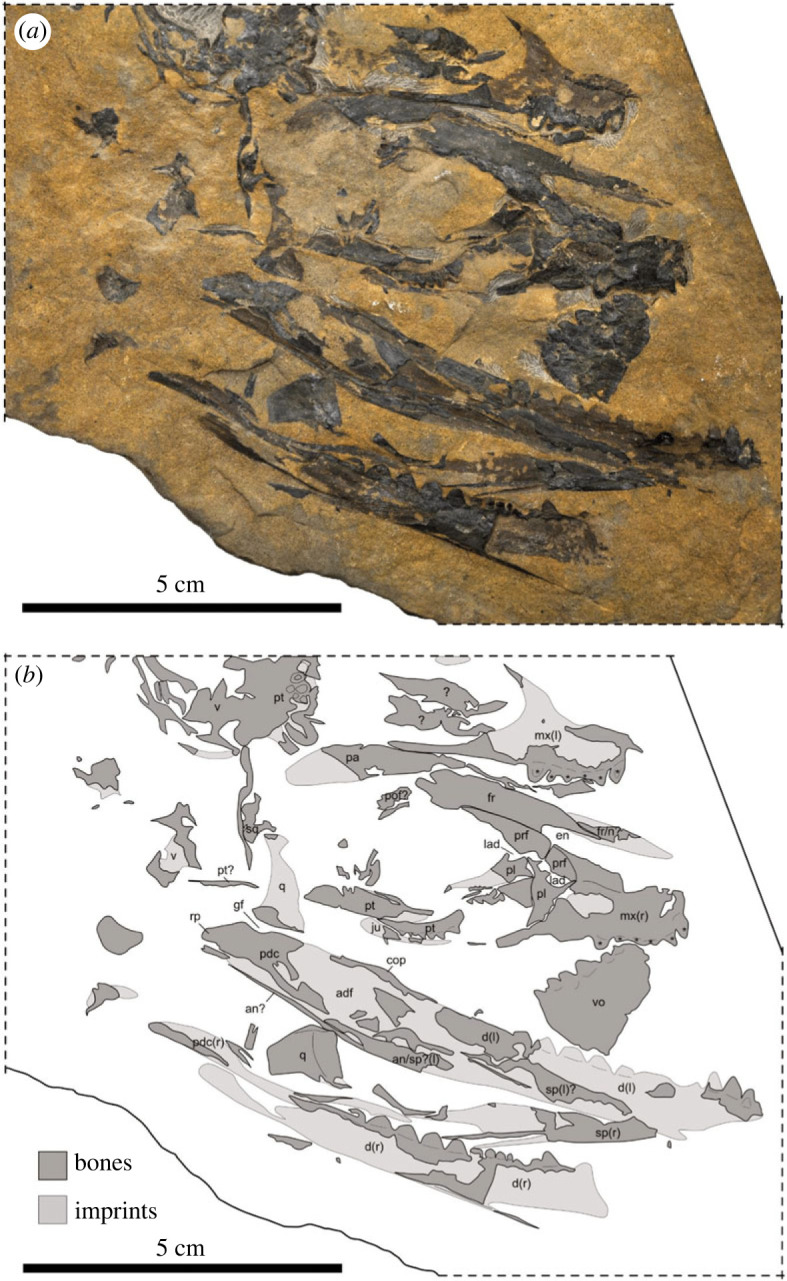
Photograph (*a*) and interpretative sketch (*b*) of the holotype TMP 89.127.1 of *Paralonectes merriami* [14]. The fossil consisting also of bone fragments and bone imprints, showing most parts of a disarticulated but associated skull mostly in right lateral view (l = left side; r = right side; note that most of associated postcranial remains are excluded here so please refer to [14] for complete specimen). **adf**, adductor fossa; **an**, angular; **cop**, coronoid process; **d**, dentary; **en**, external naris; **fr**, frontal; **gf**, glenoid fossa; **ju**, jugal; **lad**, lacrimal duct; **mx**, maxilla; **n**, nasal; **pa**, parietal; **pl,** palatine; **pdc**, postdentary complex; **pof**, postorbitofrontal; **prf**, prefrontal; **pt**, pterygoid; **q**, quadrate; **rp**, retroarticular process; **sp**, splenial; **sq**, squamosal; **v**, vertebra; **vo**, vomer; *, marginal or palatal dentition.

**Figure 4.**
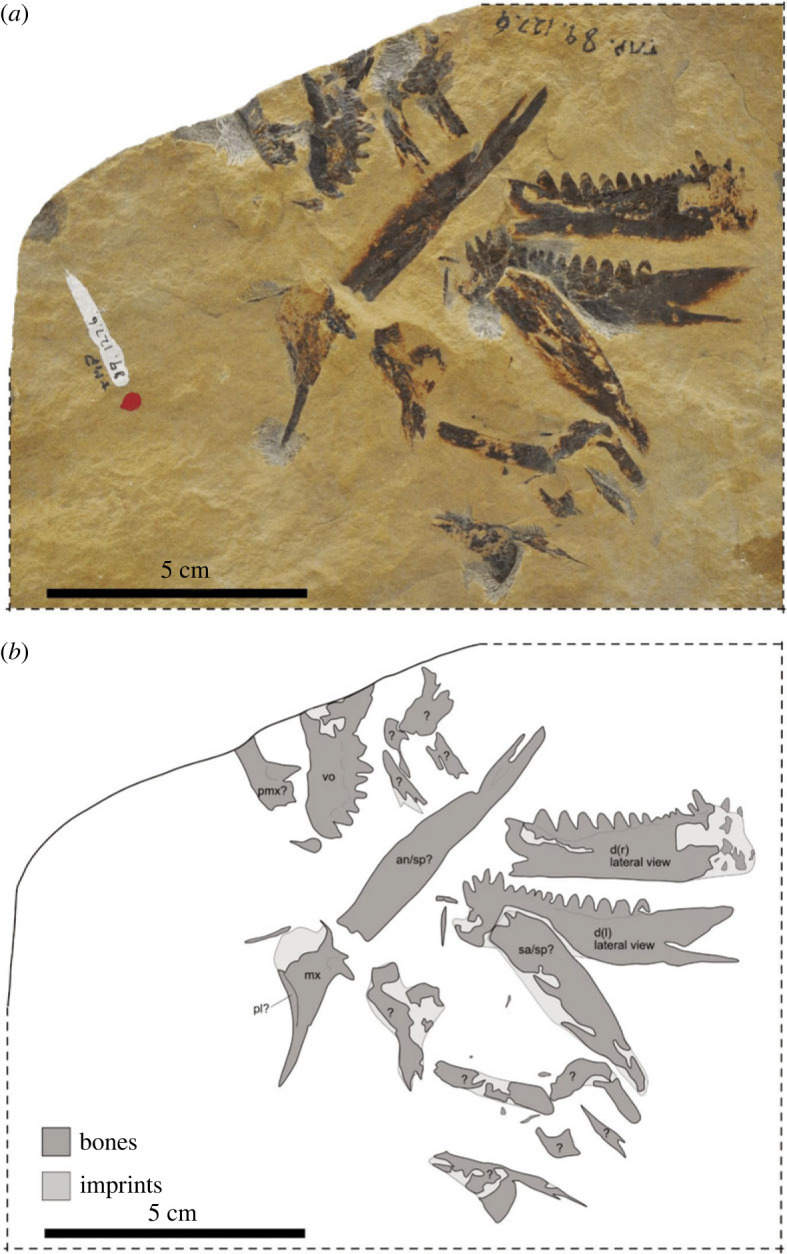
Photograph (*a*) and interpretative sketch (*b*) of the holotype TMP 89.127.6 of *Agkistrognathus campbelli* [14]. The specimen is disarticulated and only few elements can be identified herein. **an**, angular; **d**, dentary; **mx**, maxilla; **pl**, palatine; **pmx**, premaxilla; **sa**, surangular; **sp**, splenial; **vo**, vomer; *, marginal or palatal dentition.

By contrast, the supranarial process of the premaxilla reaches the frontal in all thalattosaurians (figure 3; [35]) and, at least in thalattosauroids, the transverse flange of the pterygoid bears an extensive dentigerous area ([36,37]). Among North American thalattosauroids, the high tooth count, sharp recurved apical morphology, and high number of posteromedially oriented tooth rows (≥5) on the pterygoid of TMP 86.153.14 closely resembles *T. alexandrae* (fig. 5 in [16]), while the overall pterygoid morphology is similar to *Nectosaurus halius* (e.g. UCMP 137371, fig. 17 in [16]). The relative size of the dentigerous shelf of the pterygoid compared to the orbit, comprising roughly two-thirds of the anteroposterior orbital length based on the postorbitofrontal facet on the frontal, is comparable to *Paralonectes* (TMP 89.127.1, figure 4) and *T. alexandrae* (UCMP 9085, [16]; D. Bastiaans 2022, personal observation). In addition, thalattosauroids from Wapiti Lake exhibit size ranges comparable to *Wapitisaurus*, with *T. borealis*, *P. merriami* (figure 3) and *A. campbelli* (figure 4) all exceeding cranial lengths of 100 mm [14]. As noted by [1], the (sub)thecodont tooth implantation in the posterior dentary of TMP 86.153.14, conforms with the (ankylosed) thecodonty which is prevalent among North American thalattosaurs [14,16].

The original study [1] dismissed a close relationship between *W. problematicus* and thalattosaurs based on the architecture and proportions of the postorbital skull region. The triangular and posteriorly heightening appearance of the skull, as described as a defining feature [1], is heavily influenced by the identification and assumed articulation of the same posterior cranial elements. As demonstrated here, the reinterpretation of these elements and the lack of much of the cranium posterior to the orbit, precludes statements about the dimensions of the upper temporal fenestra and overall skull proportions. Therefore, these characteristics no longer serve as distinguishing features of *Wapitisaurus* compared to thalattosaurians.

Additional skull characteristics also hint at a close relationship between *Wapitisaurus* and thalattosauroids. First, *Wapitisaurus* shows a ventral deflection of the dorsal margin of the premaxilla, resembling *Paralonectes* and *Thalattosaurus* among North American thalattosauroids (fig. 7 in [14] and fig. 14 in [16]). Second, the newly identified maxilla of the *W. problematicus* holotype only shows a short dentigerous portion that does not extend to the level of the very long posterior (jugal) process. A similarly proportioned maxilla with a short dentigerous margin can be observed in *Paralonectes* (e.g. TMP 89.127.1, fig. 7 in [14]; TMP 89.126.2; see [14]). This condition is also seen in other (or even most) later thalattosauroids including *Thalattosaurus* (fig. 1 in [16], D. Bastiaans 2022, personal observation), *Nectosaurus* (figs 18 and 19 in [16]) and *Xinpusaurus* (figs 2 and 3 in [38]). Third, the external naris of *W. problematicus* is located very far posteriorly on the rostrum and framed dorsally by a slender, crescentic nasal (figure 2*a,b*). As noted above, this morphology closely resembles that seen in thalattosauroids, as exemplified by *T. alexandrae* in which the nasal extends beyond the anterior margin of the external naris into a dorsal socket, covered by the ventral margin of the premaxilla (fig. 14 in [16] and fig. 2 in [24]). Fourth, the long, L-shaped palatine of *W. problematicus* is also comparable to that of *Thalattosaurus* (Plate III, Plate IV fig. 3 of [23]), being a gently sloping element with a dorsoventrally tall anterior section and a long horizontal posterior process forming the palatal shelf [16,24].

Several characteristics of the mandibular dentition further indicate a potential close relationship with or even inclusion of *Wapitisaurus* in thalattosauroids. First, the anterior teeth are positioned along the dorsal margin of the dentary, while the posterior teeth show a larger occupation along the medial surface of the bone. This trait is shared with *P. merriami* and is even more pronounced in *T. alexandrae* [16,24]. Second, the dentary teeth show some degree of heterodonty in terms of tooth size and gross morphology, which is a common feature observed in the mandibles of thalattosauroids, such as *Nectosaurus*, *Xinpusaurus*, *Paralonectes*, *Thalattosaurus* and *Agkistrognathus* [14,16,24,38]. Third, *Wapitisaurus* has 11–12 preserved dentary teeth. North American thalattosauroids have a tooth count ranging from 11 to 13 in *Nectosaurus* on the low end, and 17 to 18 in *T. alexandrae* [16], and intermediate values in *Paralonectes* (13–15: [14]) and *Agkistrognathus* (15: [14]). However, it is important to note that tooth count can be highly variable ontogenetically, intra- and interspecifically, and a low tooth count in *Wapitisaurus* may be a unique feature regardless of its assignment to any particular clade. Fourth, as for the maxilla, the dentigerous portion of the dentary terminates well anterior to or at the orbital rim. This morphology can also be seen in North American thalattosauroids such as *Thalattosaurus* [16], *Nectosaurus* [16] and *Paralonectes* (figure 3; [14]).

Lastly, the overall mandibular morphology of *Wapitisaurus* also conforms well to that of thalattosauroids. First, *Wapitisaurus* possesses a prominent coronoid process that extends along the dorsal margin of the mandible in the ventral part of the orbit. Most thalattosauroid species exhibit a high and pointed process with a longer anterior than posterior extent, and a wavy ventral margin as seen in *Nectosaurus* and *Thalattosaurus* ([16]; D. Bastiaans 2022, personal observation). The only noticeable difference between the coronoid process of thalattosauroids and that of *Wapitisaurus* is the angle of the dorsal process, which seems to be more anteriorly or dorsally facing in the latter. Second, in *Wapitisaurus*, the retroarticular process is positioned along the dorsal margin of the mandible, as in thalattosauroids such as *Nec. halius* [16] and *Paralonectes* [14]. Third, these two taxa, also show a posterodorsally inclined ventral mandibular margin, which is similar to *Wapitisaurus*. Fourth, in *Wapitisaurus*, the glenoid fossa is deep and has two thickened bony edges before the mandible rapidly decreases in height to form the retroarticular process. This suggests that a well-developed ventral quadratic condyle articulated with the mandible at a relatively right angle to the horizontal axis. Thalattosauroids like *Nectosaurus* [16], *Paralonectes* [14], *Xinpusaurus* [38] and even *Clarazia* [36] and *Hescheleria* [36] generally have a deep glenoid fossa, with the former three taxa having a slightly stronger posterior border and the latter two showing the opposite pattern. Overall, the ventral margin of the posterior mandible, the morphology and proportions of the glenoid fossa and retroarticular process resemble those of *Nec. halius* the most.

In light of the numerous similarities noted above, we confidently assign *W. problematicus* to the Thalattosauroidea. However, *Wapitisaurus* stands out from virtually all known North American thalattosauroids in that it has an unusually large orbit. Indeed, based on the proportions of the right hemimandible, it is likely that *Wapitisaurus* had a relatively large orbit and a restricted posterior cranium, which is uncommon in thalattosauroids but present in taxa such as *Thalattosaurus* [16,24], *Paralonectes* [14] and especially in a yet unpublished Brisbois Member thalattosauroid [39,40].

The tooth crown morphology of both the mandible and the maxilla dentition strongly differs from any known thalattosauroid in being conical with a sharp slightly (re)curved apex. The dorsally oriented coronoid process is rather unusual, as most thalattosauroids show a prominent posterodorsal curvature. In addition, the dorsal mandibular shelf is relatively straight terminating in a short but prominent retroarticular process as only comparable to the holotype of *Nec. halius* (fig. 21 in [16]), while the ventral margin exhibits a posterodorsal curvature. This posterodorsal curvature is a common feature in thalattosauroids, but the relatively straight ventral and anterior margin of the mandible in *Wapitisaurus* is rather unusual. These features may be partly emphasized by the poor preservation of these regions. Furthermore, the proportions of certain cranial elements, such as the pterygoid and palatine, being very robust and anteroposteriorly elongate, and the relatively small size of the squamosal and prefrontal compared to other thalattosauroids could potentially be explained as specific features of *Wapitisaurus*. In fact, North American and European thalattosauroids show distinct but wide spectra of relative sizes of these elements (e.g. [14,16,24,36]). Lastly, the comparably short premaxillary rostrum seems reminiscent of *T. borealis*, however, in *Wapitisaurus* the premaxilla does not seem to extend (far) below the marginal dentition.

Overall, the numerous similarities in cranial and mandibular features between *Wapitisaurus* and North American thalattosauroids suggests a close relationship between them. However, we highlighted several distinct differences in orbital, overall cranial, pterygoid and individual pre- and postorbital element dimensions (e.g. relative size of squamosal to overall skull size) between *W. problematicus* and known North American thalattosauroid taxa. In light of these differences, we here assign *W. problematicus* to the Thalattosauroidea as a distinct, valid species.

### 4.2. Implications for weigeltisaurid and thalattosauroid evolution

The previous identification of *W. problematicus* as a weigeltisaurid raised several problematic questions regarding weigeltisaurid evolution. First, as all weigeltisaurids are known from the Lopingian (possibly late Capitanian as well; [5,41]), the stratigraphic occurrence of *Wapitisaurus* in the Early Triassic extended the clade's temporal range past the end-Permian Mass Extinction (EPME). This would raise the question of how weigeltisaurids survived this catastrophic event. This was especially surprising given the severity of the EPME (e.g. [42–44]), with only few non-saurian reptiles surviving this event [45].

Second, *Wapitisaurus* is very large compared to other weigeltisaurids, and as suggested by [1], it is unlikely that its size allowed it to glide. In addition, *Wapitisaurus* is recovered in marine sediments with a fauna of marine reptiles and fishes that contrasts the palaeoenvironments in which Permian weigeltisaurids lived, which probably corresponded to a forest environment in the vicinity of a body of water in which their recovered remains have been preserved [5].

The depositional setting of *Wapitisaurus* thus does not conform well to a forested environment, which is typically the habitat favoured by terrestrial gliders [46–48], further contradicting a gliding lifestyle. If *Wapitisaurus* was a weigeltisaurid, then its size and depositional setting would represent the first putative transition from a gliding lifestyle to a terrestrial one. While loss of powered flight is known in birds, there is no case of secondary loss of flight in gliding animals [45–47].

Thus, by demonstrating that *Wapitisaurus* is not a weigeltisaurid, we clear up some confusion regarding the later evolution of the clade, both in temporal, geographical and ecological terms. Despite that, given the scarcity of weigeltisaurid-bearing localities and the imprecise age given for their Malagasy occurrence [3,5], much remains unclear of the evolution of the first gliding reptiles.

If *W. problematicus* is indeed from the Early Triassic period and can be reassigned to Thalattosauroidea, it would represent one of the oldest representatives of the clade. Being a rare exception to the historic scarcity and incompleteness of Lower Triassic thalattosaurian remains, it would significantly contribute to our understanding of their biogeographic and evolutionary origins. Unfortunately, much of the posterior cranium is missing, preventing detailed examination of the evolution and development of the upper temporal fenestrae in this taxon. This feature is one of the distinguishing characteristics that sets thalattosaurs apart from (most) other marine reptile groups. Despite this limitation, many traits commonly associated with thalattosauroids can be observed in the earliest representatives, such as *Paralonectes* and various indeterminate thalattosauroid remains from the Early Triassic. These shared characteristics include a presumably akinetic skull (based on the frontal morphology) with an incomplete lower temporal arcade; a slightly curved premaxillary rostrum; heterodont dentition with (sub)thecodont implantation that has a limited occupation along the maxillary and mandibular shelf; a robust pterygoid with numerous sharp, recurved teeth; and a deep mandible with a prominent glenoid fossa for a well-developed quadratic condyle and robust coronoid process. These features suggest a close relationship between *Wapitisaurus* and other thalattosauroids.

The body length of *Wapitisaurus* would have been between 1 and 1.5 m, based on estimates of *Thalattosaurus* (UCMP 9085) or up to 1.9 m, based on estimates for *Askeptosaurus* (PIMUZ T5391, ‘Besano II’ specimen), if proportions of cranium to body length are retained throughout Thalattosauriformes [16]. These size estimates align well with body lengths of most presumed Early Triassic thalattosaur fossils and the better represented Middle Triassic thalattosauroids.

Overall, the presence of potentially another genus of thalattosauroid in the Early Triassic of western North America and specifically British Columbia, further illustrates a previous underestimation of thalattosauroid diversity and disparity during this time driven by a historic dearth of material and limited knowledge of thalattosauroid morphology. Ongoing efforts focussing on the re-description of historical material, as well as new phylogenetic analyses of thalattosaur in-group relationships, will allow for *Wapitisaurus* to be included in a future detailed revision of North American thalattosaur systematics. The close morphological similarities with later representatives of the clade, including the posterior placement of the external nares, and their high abundance in Lower Triassic (shallow) marine settings may indicate an earlier invasion of this realm than previously assumed. These patterns parallel observations in early ichthyopterygians with widespread opportunistic trophic niche diversification in the shallow marine realm relatively rapidly after the EPME event [49].

## 5. Conclusion

*Wapitisaurus problematicus*, as it name suggests, has had a problematic status within the Weigeltisauridae ever since its erection. The holotype specimen from the lower strata of the Sulphur Mountain Formation of the Wapiti Lake area, British Columbia, Canada, has widened the spatio-temporal range of the group by representing the sole specimen of Early Triassic age and the only find outside of Eurasia and Madagascar. The poor quality and limited quantity of preserved remains, however, raised doubts repeatedly on its assignment to the weigeltisaurids. Re-examination and comparison with all known weigeltisaurids, as well as coeval reptiles from the Sulphur Mountain Formation has revealed that *W. problematicus* is not part of the clade of earliest gliding reptiles known, but instead may belong to the marine thalattosaurs. Similarities in dentary and maxilla shape and dentition, circumorbital bone arrangement, pterygoid shape and dentition resemble that of the genera *Thalattosaurus, Nectosaurus* and *Paralonectes*. This re-assignment of the taxon to the Thalattosauroidea results in (i) the Weigeltisauridae again representing a Late Permian clade, still absent from North America and (ii) an increase in the diversity of Early Triassic thalattosauroids diagnostic to sub-family level from British Columbia. *Wapitisaurus problematicus* represents one of the oldest representatives of the clade. Being a rare exception to the historic scarcity and incompleteness of Lower Triassic thalattosaurian remains, it significantly contributes to our understanding of their biogeographic and evolutionary origins. The presence of another genus of thalattosauroid, in addition to *Paralonectes* sp. and various indeterminate thalattosaur remains from Meosin Mountain (RTMP collections), illustrates a previous underestimation of early thalattosauroid diversity and disparity driven by a historic dearth of material and limited knowledge of thalattosauroid morphology. *Wapitisaurus problematicus* already exhibits many morphological characteristics of later thalattosauroids, most notably the posterior placement of the external nares, and the high abundance of thalattosauroids in Lower Triassic (shallow) marine settings may hint at an earlier invasion of this realm than previously assumed. These patterns are reminiscent of observations in early ichthyopterygians with widespread opportunistic trophic niche diversification in the shallow marine realm relatively rapidly after the EPME event (tables 1 and 2).

## Data Availability

All specimens used in this study are stored at the Royal Tyrrell Museum of Paleontology (RTMP), Drumheller, Alberta, Canada. All comparative material has been published in peer-reviewed literature. No additional data, besides those depicted here and referred published specimens, was used in this study.
